# Haplotype stacking to improve stability of stripe rust resistance in wheat

**DOI:** 10.1007/s00122-025-05045-0

**Published:** 2025-10-06

**Authors:** Jingyang Tong, Zerihun T. Tarekegn, Dilani Jambuthenne, Hannah Robinson, Madhav Pandit, Kira Villiers, Sambasivam Periyannan, Lee Hickey, Eric Dinglasan, Ben J. Hayes

**Affiliations:** 1 Queensland Alliance for Agriculture and Food Innovation, The University of Queensland, St Lucia, QLD Australia; 2 School of Agriculture and Environmental Science & Centre for Crop Health, University of Southern Queensland, Toowoomba, QLD Australia

## Abstract

**Key message:**

Genotype-by-environment interaction analysis and haplotype-level characterisation provide novel insights into the stability of stripe rust resistance. Breeding selection strategies are proposed to achieve rapid and stable genetic gains across environments.

**Abstract:**

This study investigated stripe/yellow rust (YR) responses in the Vavilov wheat diversity panel evaluated across 11 field experiments conducted in Australia and Ethiopia during 2014–2021. Genotype-by-environment interaction (GEI) was analysed using a factor analytic (FA) model. Genotype-level selection was performed with overall performance (OP) and root-mean-square deviation (RMSD), which reflected average performance and stability of YR resistance across environments, respectively. Genomic estimated breeding values (GEBV) for these traits were calculated and compared with those from a multi-trait GBLUP model with average performance represented by the mean GEBV across environments and stability by the standard deviation of GEBV across environments. The FA-based and multi-trait GBLUP GEBV had high correlations. Haplotypes with large effects on OP and RMSD were identified using the local GEBV method. Favourable haplotypes were then used for stacking in breeding simulations, using the Vavilov collection as a base. Compared to truncation selection, optimal haplotype selection (OHS) using an artificial intelligence (AI)-based algorithm achieved longer-term genetic gains for both OP and RMSD (after many generations) by initially selecting founder parents that maximised favourable haplotypes. Simulations using YR responses from diverse environments that mimicked fluctuating environmental conditions across seasons were conducted to evaluate strategies for selection of YR resistance that is stable across years. Strategies which gave most weight to OP, but some weight to RMSD were optimal in these conditions, and substantially reduced variation of performance across years. This study provides useful information for breeding cultivars with both high YR resistance and high stability of resistance across environments.

**Supplementary Information:**

The online version contains supplementary material available at 10.1007/s00122-025-05045-0.

## Introduction

Stripe/yellow rust (YR), caused by *Puccinia striiformis* f. sp. *tritici* (*Pst*), is an economically important disease threatening wheat production globally. Historically, YR was predominantly observed in cooler climates; however, the majority of global wheat-growing regions are now increasingly vulnerable to YR due to evolution of *Pst* pathotypes with adaptation to warmer climates (Hubbard et al. 2015; Bouvet et al. 2022). More than 60 countries are under threat from YR, causing economic losses of over one billion US dollars annually, with major epidemics often reported in Ethiopia, Australia, the USA, and China (Beddow et al. 2015; Savary et al. 2019). Developing YR-resistant cultivars is widely preferred as a strategy to manage the disease due to cost-effectiveness and environmental benefits (Jørgensen et al. 2017). The response of wheat genotypes to YR is influenced by environmental factors and genotype-by-environment interaction (GEI) effects, as illustrated by the multi-environment trials (MET) and analysis of variance (ANOVA) in recent studies (Bokore et al. 2017; Zhou et al. 2022; Tong et al. 2024a). As the expression of resistance can vary depending on the environmental conditions, selection of superior genotypes remains difficult, especially when there is also variation in the pathogen population across environments or seasons (Mehta et al. 2022; Verma et al. 2024). Hence, conducting GEI analysis is essential to support the selection of genotypes with stable resistance across environments and against a dynamic pathogen population. However, research in this area is limited.

The factor analytic (FA) model is now a widely preferred approach for MET analyses in plant breeding programmes since the FA structure exhibits good performance both in effectively fitting the data and in maintaining model parsimony for GEI (Smith et al. 2001, 2015; Kelly et al. 2007). A range of summaries from the FA approach can be explored to infer GEI and examine genotypes across environments, including heatmaps of estimated genetic correlations between environments and FA-structure-derived latent regression plots for measures of overall performance and stability for each genotype across environments (Smith and Cullis 2018; Tolhurst et al. 2022). This approach has recently been used to explore lodging (Dreccer et al. 2020) and heat tolerances (Coast et al. 2022) for wheat, biomass production for sorghum (Hunt et al. 2020; Oliveira et al. 2020), and yield stability for canola (Cowling et al. 2023).

Crop breeding programmes are increasingly employing genomic selection (GS) to enhance genetic gains (Bernardo and Yu 2007; Voss-Fels et al. 2019a). GS involves ranking and selecting genotypes based on genomic estimated breeding values (GEBV), derived from genome-wide marker effects estimated with training populations and prediction models (Meuwissen et al. 2001; McGowan et al. 2021). Unlike traditional marker-assisted selection (MAS), which relies on a limited number of breeder-friendly markers to introduce and stack target beneficial alleles into a single genotype, GS aims to optimise the breeding selection process for complex traits influenced by many mutations through comprehensive marker information (McGowan et al. 2021). Although purposefully stacking large numbers of desirable alleles using GS and single markers is challenging, several strategies have been proposed to identify and combine chromosome segments defined by marker-based haplotypes with beneficial genetic effects on traits of interest. For example, Kemper et al. (2012) assumed that every chromosome segment contained at least one mutation affecting the trait, effectively representing a form of ‘gene pyramiding’ adapted to the quasi-infinitesimal model of quantitative trait variation (Hayes et al. 2023). The optimal haplotype stacking (OHS) proposed by Kemper et al. (2012) utilises a genetic algorithm, a form of artificial intelligence (AI). The initial step involves calculating haplotype effects, referred to as ‘local GEBV’, and identifying beneficial haplotypes with substantial effects on the trait. Haplotype blocks (haploblocks) exhibiting high variance of haplotype effects are likely to be important genomic regions influencing the trait (Voss-Fels et al. 2019b; Weber et al. 2023; Brunner et al. 2024). Subsequently, the genetic algorithm is used to identify a set of parents whose haplotype segments can be combined to form the ‘ultimate genotype’, which maximises the overall value of the best chromosome segments across the genome (Hayes et al. 2024). The OHS approach preserves more useful genetic diversity compared to truncation-based GS (Villiers et al. 2024) and is now being used to accelerate wheat yield improvement, with potential applications for disease resistance as well.

A historically significant wheat germplasm collection is preserved at the N.I. Vavilov Institute of Plant Genetic Resources (VIR) (Mitrofanova 2012). Previous studies have demonstrated that the ‘Vavilov wheat diversity panel’, a diverse subset of accessions, provides a valuable source of genetic resistance to rust diseases (Riaz et al. 2018; Jambuthenne et al. 2022).

Our current study aims to use the Vavilov wheat diversity panel and multi-environment trial data from 11 field experiments conducted in Australia and Ethiopia from 2014 to 2021 to: (1) explore GEI effects on YR resistance, and identify genotypes with high performance and stability for YR resistance across Australian and Ethiopian environments; (2) characterise haplotypes associated with YR resistance and stability; and (3) evaluate selection strategies with breeding simulations to achieve rapid improvement in YR resistance that is stable across diverse environments or potentially fluctuating pathotype variation.

## Materials and methods

### Plant materials and phenotypic data

This study used the Vavilov wheat diversity panel which included 295 bread wheat accessions constituted from landraces, cultivars, and breeding lines collected between 1922 and 1990 (Table S1; Riaz et al. 2017). These entries were originally sourced from 28 different countries, representing high genetic diversity.

We investigated the YR response of the Vavilov diversity panel in 11 field experiments conducted in Australia and Ethiopia from 2014 to 2021 (Table S2). The field trials were conducted and described in our previous study (Jambuthenne et al. 2022) to evaluate YR response in artificially inoculated disease nurseries in Queensland (QLD) (27°55′S, 152°34′E) over three consecutive cropping seasons (2014–2016), which are hereafter referred to as YR_14QLD, YR_15QLD, and YR_16QLD, respectively. In QLD, each annual trial was conducted at a single location using a completely randomized design, and phenotypic data were collected at two to three time-points per year (Jambuthenne et al. 2022). Eight field trials were conducted to examine the Vavilov wheat accessions in Ethiopian environments under natural pathogen infection, and detailed descriptions on Ethiopian experimental design could be found in Tarekegn (2024). The trials were performed in Meraro (7°41′N, 39°25′E) (2018, 2019 and 2021), Kulumsa (8°01′N, 39°15′E) (2018, 2019 and 2021), and Bekoji (7°35′N, 39°10′E) (2019 and 2021), which are hereafter referred to as YR_18MR, YR_19MR, YR_21MR, YR_18KU, YR_19KU, YR_21KU, YR_19BK, and YR_21BK, respectively. These studies used pathotypes that were highly virulent and prevalent across the Australian or Ethiopian wheat-belts. Disease scores assessed at the adult plant stage were assigned to each accession using the scale 0–9, where 0 is highly resistant and 9 is highly susceptible (Riaz et al. 2018; Jambuthenne et al. 2022). The conversion between the disease score and coefficient of infection is provided in Table S3.

### Multi‑environment trial analyses

Two-stage analysis of the disease data was conducted using ASReml-R (Butler et al. 2017) in R v4.2.1: (1) single-environment analysis, and (2) multi-environment trial analysis. Given that the disease score is ordinal, the assumptions of normality and homoscedasticity are justified only when the model residuals approximate a normal distribution. In the first stage, linear mixed models (LMMs) for each individual environment were fitted as follows:

$$y_{ij} = \mu + g_{i} + b_{j} + e_{ij}$$

where $y_{ij}$ represents the disease score, $\mu$ is the overall mean, $g_{i}$ is the fixed genotype effect, $b_{j}$ is the random block effect, and $e_{ij}$ is the residual error. $b_{j}$ was omitted from the model for the Australian environments.

In the second stage, the best linear unbiased estimates (BLUEs) obtained from the first stage were combined and incorporated into a multi-environment LMM to capture GEI through complex variance–covariance structures. The LMM fitted across environments was as follows:

$${\mathbf{y}} = \bf{1}_{n} \mu + {\mathbf{X\tau }} + {\mathbf{ge}} + {{\varvec{\upvarepsilon}}}$$

where ${\mathbf{y}}$ is the $n_{g} \times n_{e}$-vector of disease phenotypes (BLUEs) combined in the MET, $\bf{1}_{n}$ and $\mu$ is the *n*-vector of ones and the overall mean, ${{\varvec{\uptau}}}$ is the $n_{e}$-vector of environmental effects associated with the $n \times n_{e}$ design matrix ${\mathbf{X}}$, ${\mathbf{ge}}$ is the $n_{g} \times n_{e}$-vector of genotype effects in environments, and ${{\varvec{\upvarepsilon}}}$ ~ N (0, **R** ⨂ **E**) is the combined vector of random residuals from all trials. **R** is a diagonal matrix used as an approximate weighting matrix, defined as **R** = diag ($\frac{1}{{w_{1} }}, \frac{1}{{w_{2} }},$ …$, \frac{1}{{w_{n} }})$, wherein $w_{i}$ is set as $\frac{1}{{\left[ {{\text{SE}}\left( {{\text{BLUE}}_{i} } \right)} \right]^{2} }}$. **E** is the error variance–covariance matrix among trials and was estimated using ASReml-R.

A series of models with different variance structures were investigated to determine the optimal MET model. Initially, a diagonal variance model was fitted to ${\mathbf{ge}}$, allowing for heterogeneous genetic variances for each environment. This served as a baseline model assuming independence of GEI effects between environments. Subsequently, correlation model with homogenous variation (corv), heterogeneous correlation model (corh), and the FA model (Smith et al. 2001) were each fitted. The FA model with *k* factors was as follows:

$$\begin{aligned} {\mathbf{ge}} = & \left( {\lambda _{1} ~ \otimes ~I_{m} } \right)f_{1} + ~\left( {{\lambda }_{2} ~ \otimes ~I_{m} } \right)f_{2} + \ldots + ~(\lambda _{k} ~ \otimes ~I_{m} )f_{k} + \delta \\ = & \left( {\Lambda ~ \otimes ~I_{m} } \right)f~ + ~\delta \end{aligned}$$

where ${{\varvec{\uplambda}}}_{k}$ is the vector of environment loadings for the *k*^th^ common factor and the environment $m$, ${\mathbf{I}}_{m}$ represents an identity matrix, ${\mathbf{f}}_{k}$ is the vector of genotype scores for *k*^th^ factor, and ${\varvec{\delta}}$ is the vector of residuals.

The number of hypothetical factors (*k*) applied within this structure was increased until the best model was identified based on a holistic assessment of Akaike Information Criteria (AIC), Log-likelihood, and the percentage of genetic variance accounted for by the factors (%VAF). Genetic variance was mainly sourced from GEI, and %VAF was defined as %VAF = $\frac{{\mathop \sum \nolimits_{k} \lambda_{k}^{2} }}{{\mathop \sum \nolimits_{k} \lambda_{k}^{2} + \sigma_{\delta }^{2} }}$. The chosen MET model was utilised to obtain estimates of the GEI effects and genetic correlations between environments (Smith et al. 2001). The genetic correlations indicate the presence or absence of GEI, with high positive correlations between environments corresponding to low GEI.

Additionally, a traditional three-way LMM explicitly taking ‘location’ and ‘year’ into account was developed. In this model, ‘location’ and ‘genotype’ were fitted as fixed main effects, and ‘year’, ‘year × location’, ‘year × genotype’, and ‘year × location × genotype’ interactions were fitted as random effects.

### Exploring stability for stripe rust resistance across environments

According to Smith and Cullis (2018), the measures of overall performance (OP) and root-mean-square deviation (RMSD) were employed to represent the average performance and stability of YR resistance across environments, respectively. OP was the genotype score for the first latent factor (F_1_) of the FA model, while RMSD, which represented stability, was derived from the regression line associated with latent variable F_1_. The two indices for each genotype *i* were calculated as follows (Smith and Cullis 2018):

$${\text{OP}}_{i} = \overline{\lambda }_{1} f_{1i}$$

where $\overline{\lambda }_{1}$ is the mean environment loading for F_1_, and $f_{1i}$ is the slope (score) for genotype *i* and *F*_1_.

$${\text{RMSD}}_{i} = \sqrt { \left( {\mathop \sum \limits_{r = 2}^{k} \lambda_{rm} f_{ri} } \right)^{2} /n_{m} }$$

where $\lambda$ is the loading for environment *m* and factor *r*, $f_{ri}$ is the score for genotype *i* and factor *r*, and $n_{m}$ is the number of environments.

For YR resistance, lower values of OP and RMSD represent better performance and stability of genotypes across environments, respectively. To calculate GEBV for OP and RMSD, 34,899 genome-wide markers from Tong et al. (2024b) and a GBLUP model (VanRaden 2008) were employed.

These GEBV were compared with GEBV from a multi-trait GBLUP (MT-GBLUP) model, where each environment was fitted as a separate trait. For comparison with OP, the average of the GEBV across environments from the MT-GBLUP model was used. For comparison with RMSD, the standard deviation of the GEBV across environments was used. Spearman rank correlations were computed between GEBV calculated using the FA-based and MT-GBLUP approaches. The MT-GBLUP model with *n*_*m*_ environments (traits) was fitted as follows:

$${\mathbf{y}} = \bf{1}_{n} \mu + {\mathbf{Zu}} + {{\varvec{\upvarepsilon}}}$$

where ${\mathbf{y}}$ are the disease phenotypes, $\mu$ is the overall mean, ${\mathbf{Z}}$ is the design matrix, ${\mathbf{u}}$ are the random breeding values ~ N (0, ${\mathbf{G}}{ } \otimes { }{\mathbf{H}}$), ${\mathbf{G}}$ is the genomic relationship matrix (GRM) (constructed following VanRaden (2008)) and ${\mathbf{H}}$ is the genetic variance–covariance matrix among traits, and ${{\varvec{\upvarepsilon}}}$ are the random residuals ~ N (0, ${\mathbf{R}}{ } \otimes { }{\mathbf{E}}$), where **E** is error variance–covariance matrix among *m* environments (traits), and **R** is a weighting matrix, defined as **R** = diag ($\frac{1}{{w_{1} }}, \frac{1}{{w_{2} }},$ …$, \frac{1}{{w_{n} }})$, wherein $w_{i}$ is set as $\frac{1}{{\left[ {{\text{SE}}\left( {{\text{BLUE}}_{i} } \right)} \right]^{2} }}$. The weighting approach ensures that observations with higher precision have a proportionally greater influence on prediction. **H** and **E** were estimated with ASReml-R.

### Local GEBV calculation and haploblock discovery

To calculate local GEBV (Fan et al. 2011; Villiers et al. 2024), 19,725 genome-wide haploblocks constructed in Tong et al. (2024b) were used, where a linkage disequilibrium (LD) threshold of pairwise *r*^2^ ≥ 0.5 between markers was adopted to define haploblocks across all chromosomes. Local GEBV for haplotypes within each haploblock for OP and RMSD of YR resistance was calculated as follows (Villiers et al. 2024):

$${\text{Local GEBV}} = \mathop \sum \limits_{t \in f} h_{t} \alpha_{1t} + \left( {1 - h_{t} } \right)\alpha_{0t}$$

where $f$ is the set of markers belonging to a haplotype/haploblock, $h_{t}$ is the allele of one marker (0 or 1), and $\alpha_{0t}$ and $\alpha_{1t}$ are the additive marker effects of alleles 0 and 1 on marker *t*. The marker effects were obtained by backsolving the GEBV estimated for each genotype for OP and RMSD, using the following equation (Strandén and Garrick 2009):

$$\hat{\boldsymbol{\upalpha} } = {\mathbf{M}}^{\prime}{\mathbf{G}}^{ - 1} {\hat{\mathbf{v}}}/\mathop \sum \limits_{t = 1}^{l} 2p_{t} \left( {1 - p_{t} } \right)$$

where $\hat{\boldsymbol{\upalpha} }$ is the vector of estimated marker effects, ${\mathbf{M}}$ is the allele matrix for all markers, ${\mathbf{G}}$ is the GRM, ${\hat{\mathbf{v}}}$ is the vector of GEBV, *l* is the number of markers, and $p_{t}$ is the allele frequency of the *t*^th^ marker.

To identify the haploblocks potentially associated with OP and RMSD of YR resistance across environments, the variances of the local GEBV were calculated in each block and scaled to a 0–1 range (Voss-Fels et al. 2019b; Brunner et al. 2024). The top ten haploblocks with the highest variances and scaled variances exceeding 0.9 were considered to be the most important trait-associated genomic regions (Voss-Fels et al. 2019b; Brunner et al. 2024).

### Breeding simulations in different environments

The simulations did not aim to fully simulate a complex real-life breeding programme but rather provide a baseline for understanding and deriving selection strategies to rapidly enhance YR resistance when environments or pathotypes fluctuate over time. The structure of the breeding simulation experiment is shown in Fig. S1. Fifty founder parents were selected using either truncation genomic selection (TS) or OHS from the Vavilov wheat diversity panel. TS selected the parents with the best GEBV for the target trait, while OHS selected parents containing haplotype segments to produce the highest possible scoring ‘ultimate’ genotype (Kemper et al. 2012; Villiers et al. 2024). OHS can select a set of parents through a genetic algorithm, a search method based on Darwin evolutionary theory. These selected parents maximise the overall value of the best chromosome segments across the genome. In this study, the fitness function that OHS optimised was:

$$\mathop \sum \limits_{i = 1}^{50} \mathop \sum \limits_{c = 1}^{19725} s_{ic} d_{ic}$$

where $d_{ic}$ is the haplotype effect of the better chromosome segment at position *c* from genotype *i*, $s_{ic}$ is a set of binary (0 or 1) decision variables, wherein 0 indicates non-target segment and 1 is for target segment. The number of parents to be selected is 50.

A genetic algorithm produced by Kemper et al. (2012) was employed to iteratively refine solutions toward optimal selection. Further information on the genetic algorithm implementation design is available in the Supporting Information of Kemper et al. (2012). Briefly, the process began with an initial population of 500 randomly generated groups of 50 candidate individuals. In each iteration, the fitness of each group was evaluated based on the predefined fitness function, which quantified the desirability of the selected individuals based on their chromosome segment contributions. Each iteration included the procedures of roulette wheel selection, crossover, and mutation. The fitness function reliably converged within 1,000 generations of iteration. The group with the highest final fitness score was used as the set of founder parents for subsequent breeding simulations.

F1 progenies were produced by half-diallel crosses, followed by selecting 10, 25, or 50 genotypes with the best GEBV as parents in the next cycle or generation, with progeny gametes sampled from parents using the program genomicSimulation (Villiers et al. 2022). In total, 100 cycles were carried out to test the long-term selection effects. Three sets of stochastic simulations were performed using genomicSimulation to provide insights into the best selection strategies with stable or fluctuating environments:

1. Improving both OP and RMSD traits of YR resistance with a stable environment (combined Australian and Ethiopian environments);

(2) The impact of selecting in an average environment (average of Australia and Ethiopia) on performance in each environment (Australia or Ethiopia);

(3) Selection in a location where the environment fluctuated between years (with Australian or Ethiopian environments simulated in alternate years), to mimic a production scenario that experiences large fluctuations in environmental conditions or pathotypes across seasons (with Australian and Ethiopian environments used to mimic two extreme environments).

For (1), breeding simulations were performed using sets of marker effects estimated from the Vavilov reference population in combined Australian and Ethiopian environments. Three different selection strategies were used, (a) selecting (with either OHS or TS) on GEBV OP of YR resistance, with the change in both OP and RMSD investigated based on the GEBV for these traits in each generation; (b) selecting (with either OHS or truncation selection) on RMSD, with the change in both OP and RMSD investigated, based on the change in GEBV for these traits; and (c) selecting on a new index (SI) developed for both OP and RMSD as follows:

$${\text{SI}} = 50\% \frac{OP}{{\sigma_{OP} }} + 50\% \frac{RMSD}{{\sigma_{RMSD} }}$$

where the weights of OP and RMSD are each set to 50%, and $\sigma_{OP}$ and $\sigma_{RMSD}$ are the standard deviations of OP and RMSD, respectively, and either OHS or TS on index was used to select parents.

In terms of purpose (2), TS- and OHS-selected founder parents based on the selection index described above were used in three simulated breeding scenarios: (a) SI-based selection in Australia; (b) Ethiopia; and (c) combined Australian and Ethiopian environments to predict changes in OP and RMSD based on GEBV in Australia and Ethiopia.

To explore the effects of fluctuating conditions (e.g. distinct weather and climate situations, changing pathotypes in different years) on OP and RMSD of YR resistance, we assumed that the breeding selections occurred in a region with environmental conditions alternating between those characteristics of Australia and Ethiopia. Figure S2 shows some examples of environmental data in Australian and Ethiopian locations, suggesting that there is significant difference in weather conditions between Australia and Ethiopia. Accordingly, we assumed in the scenario (3) environmental characteristics alternated from year to year between that of Australia and Ethiopia. In this scenario, in breeding generation 1, GEBV1 was the GEBV for OP of YR resistance in Australia, and true breeding value 1 (TBV1) in Australia was calculated from random sampling from a bivariate normal distribution given GEBV1, where the correlation between GEBV1 and TBV1 was assumed to be 0.5 to represent the accuracy of GEBV1 in Australia. The progenies with the best TBV1 for OP were selected. In breeding generation 2, GEBV2, the GEBV for OP in Ethiopia, was calculated from random sampling from a bivariate normal distribution given GEBV1, where the correlation between GEBV1 and GEBV2 was the genetic correlation of OP between Australia and Ethiopia. Then true breeding value 2 (TBV2) in Ethiopia was obtained by random sampling from a bivariate normal distribution given GEBV2, where the correlation between GEBV2 and TBV2 was 0.5. This procedure was repeated until generation 100. In another simulation, SI was used for selecting the best candidates in the aforementioned breeding scenario, and different combinations of weights (i.e. 90, 80, 70, 60 or 50% OP + 10, 20, 30, 40 or 50% RMSD) were tested to optimise the SI.

## Results

### MET analysis reveals significant GEI patterns for YR resistance across Australia and Ethiopia

**Fig. 1:**
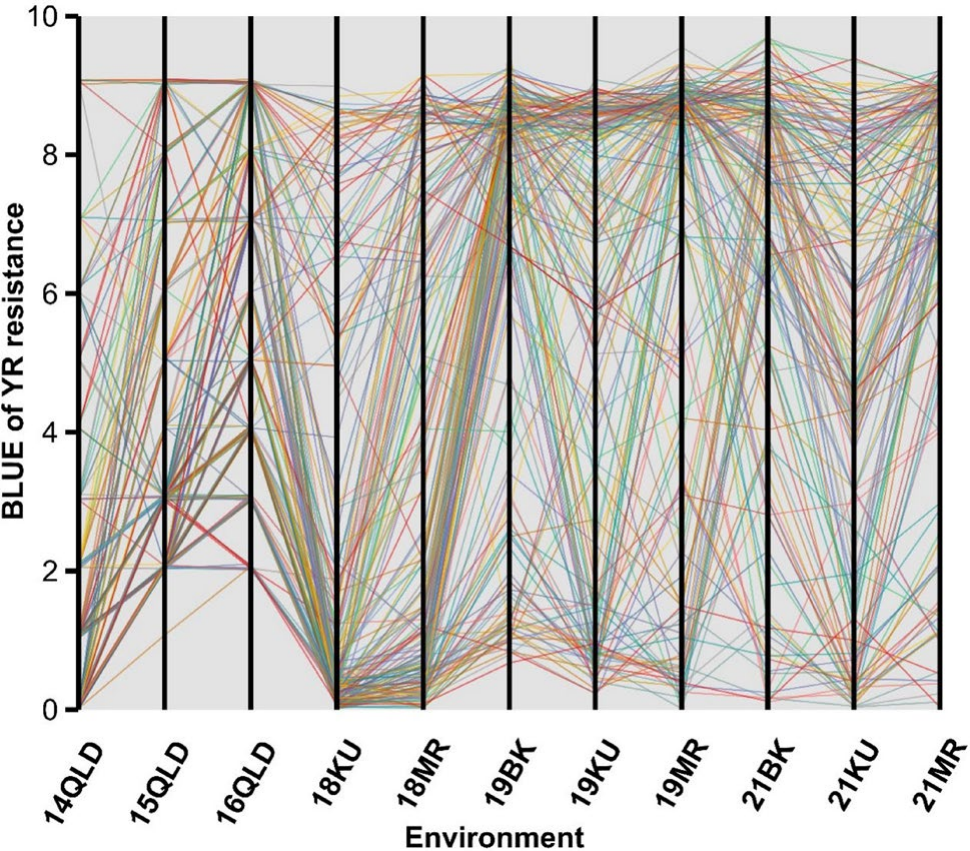
Single-environment analysis for 295 Vavilov wheat accessions using 11 field phenotypic datasets collected for YR resistance in Australia and Ethiopia. QLD, Queensland; MR, Meraro; KU, Kulumsa; BK, Bekoji

**Fig. 2:**
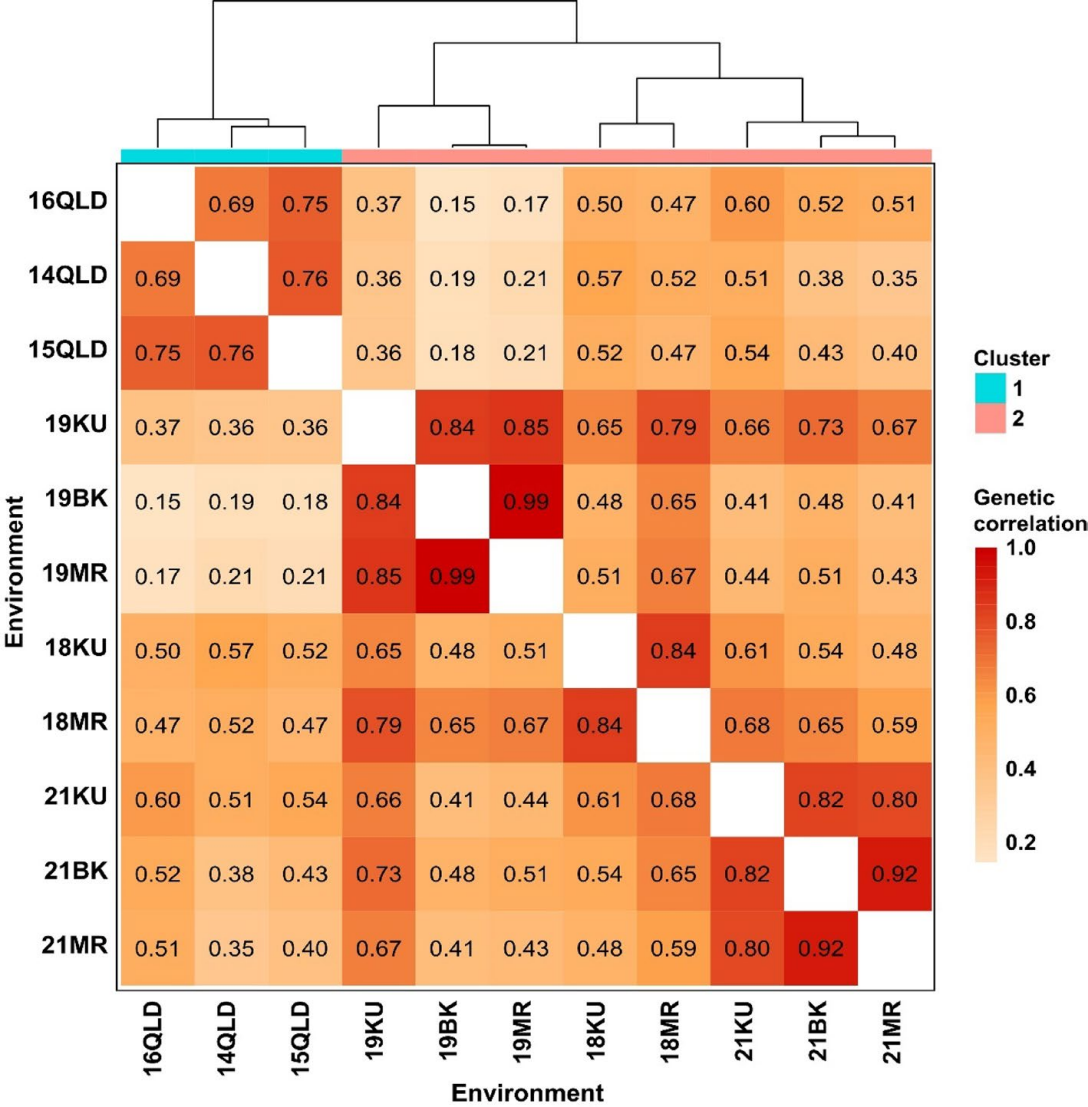
Genetic correlation matrix for YR resistance for 11 environments, ordered by Ward’s clustering. Estimated correlation coefficients for pairs of environments are denoted within the cells. QLD, Queensland; MR, Meraro; KU, Kulumsa; BK, Bekoji

A wide range of variability was observed for YR resistance among the Vavilov wheat accessions in Australian and Ethiopian environments (Fig. 1; Fig. S3). Different genotype rankings and significant crossovers between these environments strongly suggested the presence of crossover GEI for YR resistance. The FA_4_ model (i.e. the FA model with four factors) had the smallest AIC (5153) and Log-likelihood (-2528), but the largest %VAF (88.3%) (Table 1). Therefore, it was selected as the best MET model. The genetic correlations indicated the presence of GEI among many, but not all environments (Fig. 2). High positive correlations between environments corresponded to low GEI, i.e. high correlations meant genotypes had similar YR resistance rankings across multiple environments. Two distinct breeding zones, Australia and Ethiopia, were clearly defined based on Ward’s clustering shown in the correlation heatmap (Fig. 2). A relatively high degree of correlation was observed within breeding zones (Australia or Ethiopia) compared to between breeding zones. In the same year, genetic correlations of YR resistance were very high (*r* = 0.80–0.99) among different locations in Ethiopia, indicating low GEI between Ethiopian locations.

**Table 1:** Goodness-of-fit statistics for the Diagonal variance model, Correlation model with homogenous variation (CorV) or heterogeneous variation (CorH), and Factor Analytic (FA) models of increasing order from one to four ^a^

Model	AIC	Log-likelihood	%VAF
Diagonal	6863	−3421	/
CorV	6008	−3002	/
CorH	5281	−2584	/
FA_1_	5784	−2870	63.1
FA_2_	5384	−2661	77.5
FA_3_	5209	−2565	85.0
FA_4_	5153	−2528	88.3

^a^AIC, Akaike Information Criteria; %VAF, percentage of genetic variance accounted for

The rotated factor loadings and %VAF from the FA_4_ model for each individual environment are shown in Table 2 to facilitate model interpretation. The high %VAF contributed by all four factors in each environment indicated a good fit of the model for GEI. The lowest accumulated %VAF, at 72.7%, was observed at 14QLD, while the highest, up to 100%, was at 19BK (Table 2). A large positive or negative loading value indicated a strong sensitivity of the environment to the underlying or unknown factor. For factor 1, which accounted for the most genetic variance of YR resistance (%VAF: 28.0–82.7%), Australian environments were significantly distinct from others, with very low loadings ranging from 0.932 to 1.065. In contrast, Ethiopian environments had loadings all exceeding 2.000. These results were consistent with earlier genetic correlation and cluster analyses, which showed that Australian environments were distinct from Ethiopian environments (Fig. 2).

**Table 2:** Restricted maximum likelihood (REML) estimates of rotated factor loadings and VAF% from the FA_4_ model ^a^

Environment	Rotated factor loadings					%Variance accounted for				
	Factor1	Factor2	Factor3	Factor4		Factor1	Factor2	Factor3	Factor4	Sum of factors
14QLD	0.932	− 0.746	0.735	0.538		28.0	18.0	17.4	9.3	72.7
15QLD	0.990	− 0.888	0.631	0.834		28.3	22.7	11.5	20.0	82.5
16QLD	1.065	− 1.035	0.343	0.634		30.1	28.4	3.1	10.6	72.3
18KU	2.291	− 0.416	1.138	− 0.609		60.2	2.0	14.9	4.3	81.4
18MR	2.900	− 0.048	0.827	− 0.717		79.7	0.0	6.5	4.9	91.1
19BK	2.086	1.622	− 0.056	0.372		61.1	36.9	0.0	2.0	100.0
19KU	2.718	0.638	− 0.221	0.002		82.7	4.6	0.5	0.0	87.9
19MR	2.513	1.810	− 0.041	0.422		64.1	33.2	0.0	1.8	99.1
21BK	2.259	− 0.756	− 1.074	− 0.085		69.0	7.7	15.6	0.1	92.5
21KU	2.362	− 1.049	− 0.439	− 0.011		64.2	12.7	2.2	0.0	79.1
21MR	2.017	− 0.85	− 1.189	− 0.077		60	10.6	20.8	0.1	91.6

^a^QLD, Queensland; MR, Meraro; KU, Kulumsa; BK, Bekoji

### Selection of Vavilov accessions with broad or specific adaptation to rust environments using the FA and MT-GBLUP approaches

**Fig. 3:**
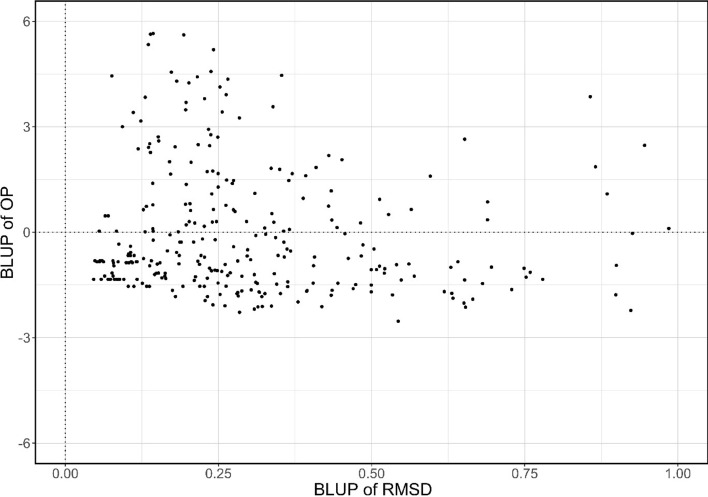
Overall performance (OP) versus root-mean-square deviation (RMSD) for stripe rust (YR) resistance of the Vavilov wheat accessions evaluated in combined environments of Australia and Ethiopia. Genomic estimated breeding values (GEBV) were calculated for these traits. Lower values of OP and RMSD represent better average performance and stability of YR resistance across environments, respectively

The Vavilov wheat accessions were evaluated for OP and RMSD of YR resistance in all environments (Fig. 3). FA-model-derived OP and RMSD represented the average performance and stability of a genotype in response to environmental changes (Smith and Cullis 2018), respectively, with lower values indicating better performance for YR resistance scores in this study. GEBV of OP and RMSD were used for selecting the superior genotypes from the Vavilov collection. Stable genotypes (low RMSD values) with high YR resistance (low OP values) were identified across all environments, such as WLA_236, WLA_192, and WLA_174 (Table S6). These lines could be considered to have broad adaptability (for rust resistance) to both Australian and Ethiopian environments. Genotype-level selection was also conducted to identify those adapted to specific environmental groups, i.e. with high specificity to either the Australian or Ethiopian breeding zones (Fig. S5). For breeders, ten wheat accessions with the highest OP or RMSD were selected from the Vavilov panel, including WLA_186 with an OP score of -1.921 in Australia and WLA_151 with an OP score of -2.404 in Ethiopia (Table S6).

**Fig. 4:**
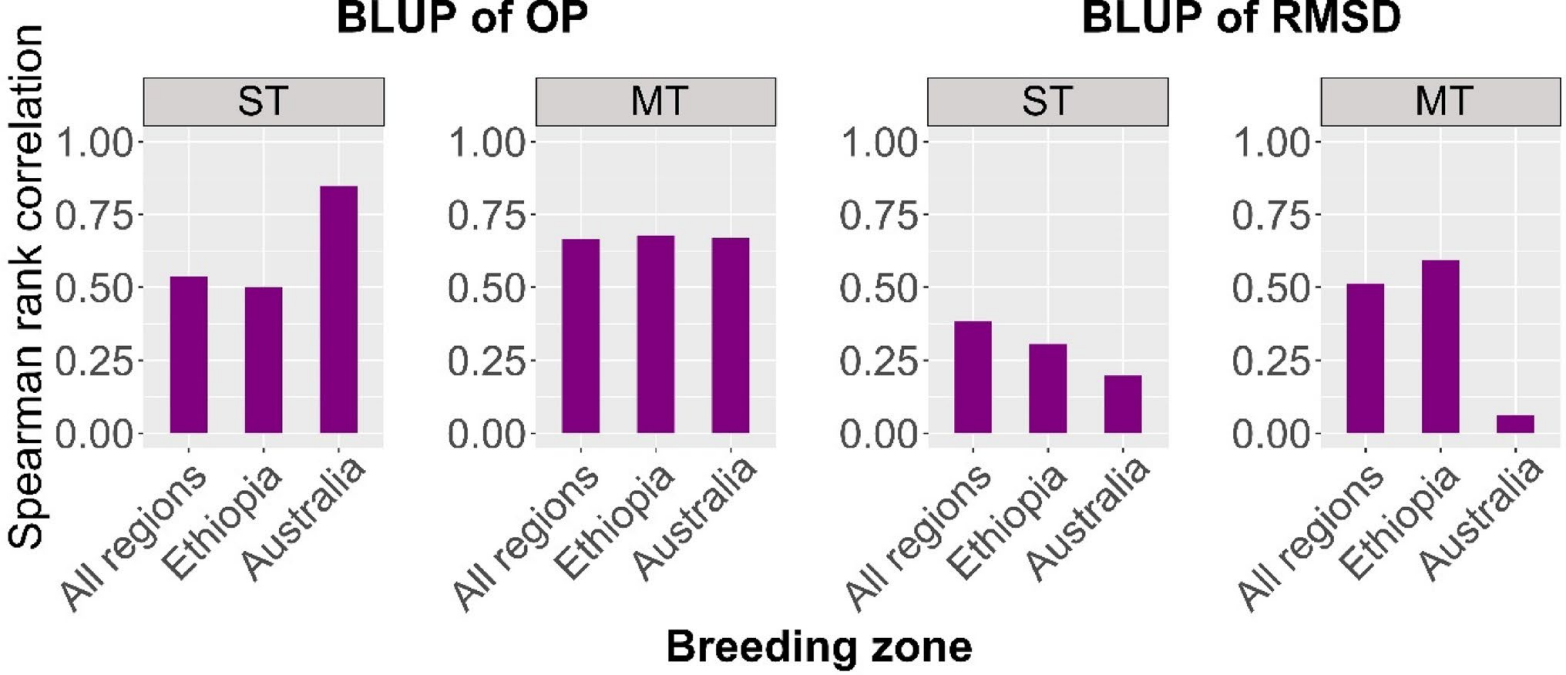
Spearman rank correlations between GEBV calculated from the single-trait GBLUP (ST-GBLUP), multi-trait GBLUP (MT-GBLUP), and factor analytic (FA)-based approaches. In ST-GBLUP and MT-GBLUP, average performance of YR resistance across environments was the average GEBV across environments, and stability was the standard deviation of GEBV across environments. The resultant average performance (the left two plots) and stability (the right two plots) were compared with the BLUP of overall performance (OP) and root-mean-square deviation (RMSD) derived from the FA model, respectively. ST, single-trait GBLUP model; MT, multi-trait GBLUP model

The GEBV of OP and RMSD of YR resistance were compared with those from a multi-trait GBLUP (MT-GBLUP) model, which provided average performance (average GEBV across environments) and stability of YR resistance (standard deviation of GEBV across environments), to assess whether the simpler multi-trait model was comparable to the relatively complex FA model in selecting superior genotypes. Spearman rank correlation analysis showed that the average GEBV of YR resistance across multiple environments (from MT-GBLUP) had high correlations (*r* = 0.64–0.68) with the GEBV of OP from the FA model (Fig. 4). The correlations between the stability of YR resistance from the MT-GBLUP model and RMSD ranged from 0.52 to 0.60, except in Australian environments, where the correlation was almost zero.

### Haplotype-level characterisation of the Vavilov diversity panel discovers genomic regions associated with YR resistance and stability

**Fig. 5:**
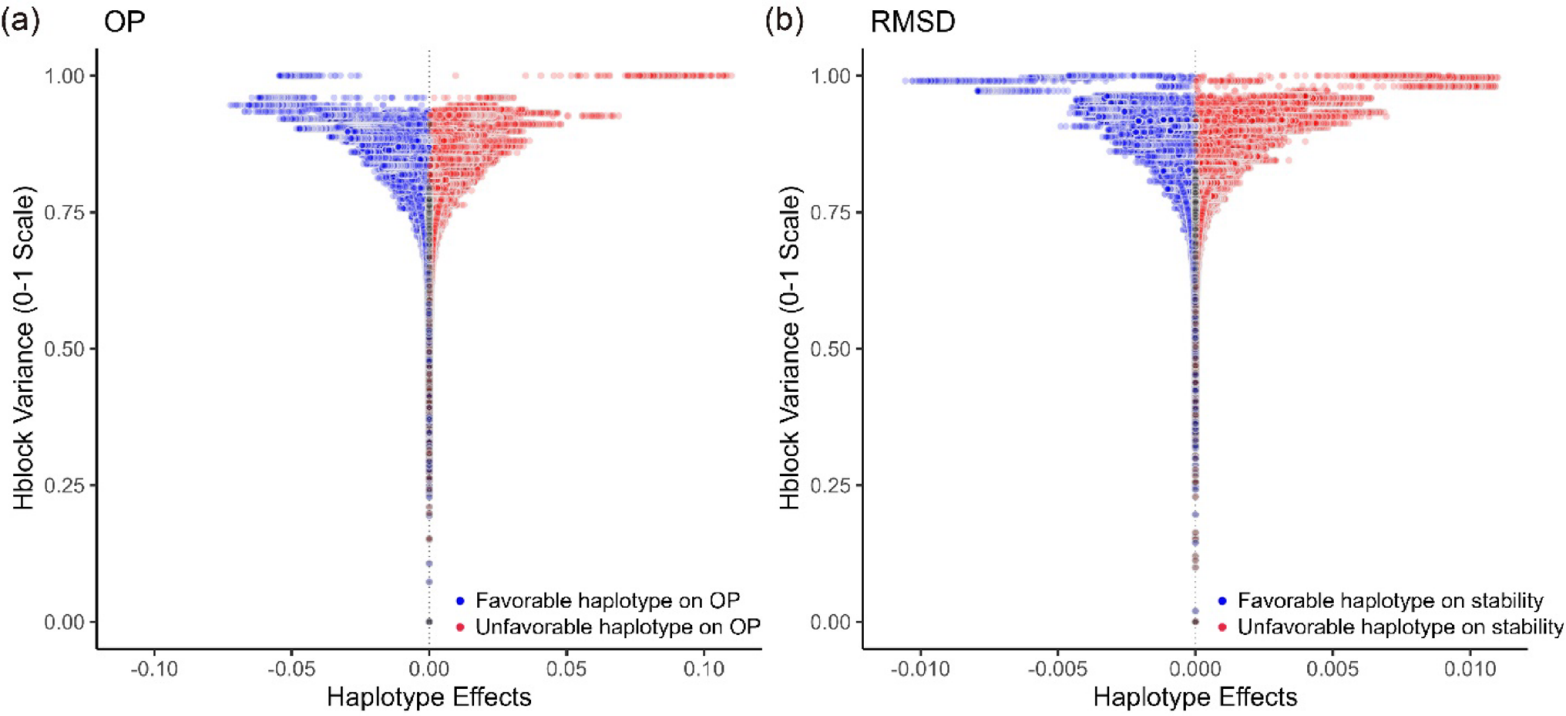
Scaled haploblock variance versus haplotype effect for each haploblock for (a) overall performance (OP) and (b) root-mean-square deviation (RMSD) of stripe rust resistance in combined environments of Australia and Ethiopia. Each dot represents a unique haplotype within the haploblock in the Vavilov wheat diversity panel, and the blue ones denote favourable resistance haplotypes, while the red denote unfavourable susceptibility haplotypes

A small proportion of haplotype effects were moderate to large for both OP and RMSD (Fig. 5). The distributions of resistant (i.e. those with negative effects) and susceptible (i.e. those with positive effects) haplotypes were nearly symmetric. The top ten haploblocks with the highest variances in haplotype effects for both OP and RMSD of YR resistance in combined Australian and Ethiopian environments are identified in Table 3. The OP-relevant genomic regions were located on chromosomes 2A (2), 2B (3), 5A (2), 5B, and 6A (2). Eight of these regions co-located with known QTL underlying YR resistance. However, b003469 and b014220, located at 97.05–106.57 Mb on 2A and 66.60–76.64 Mb on 6A, did not contain any known *Yr* genes or QTL and could therefore be considered novel. Seven of the ten haploblocks associated with RMSD contained known YR resistance QTL located on chromosomes 1A, 2A (2), 4B, 5A (2), and 6B (Table 3). In particular, b015121, located on the short arm of chromosome 6B (52.74–55.53 Mb), was in close proximity to *Yr36*, a broad-spectrum and adult-plant YR resistance gene (Fu et al. 2009). Three haploblocks, b003108 (1D, 494.81–497.25 Mb), b010090 (4A, 650.49–656.83 Mb), and b011005 (4B, 617.02–618.90 Mb), were not found to co-occur with any reported QTL related to YR and might represent novel genetic loci. Additionally, there was no overlap between the top ten haploblocks associated with OP and RMSD traits; in other words, no identified locus was associated with moderate to large effects for both traits simultaneously (Table 3). High-variance haploblocks and their favourable haplotypes could be preferentially targeted for stacking to improve YR resistance and stability of YR resistance in breeding programmes (Fig. 5).

**Table 3:** Top ten haploblocks with the highest variance of haplotype effects for OP and RMSD of stripe rust resistance in all environments^a^

Trait	Hblock	Chrom	Location (Mb)^b^	Number of markers included	Scaled variance^c^	Co-location of reported QTL/ genes related to stripe rust^d^	Reference
OP	b003469	2A	97.05–106.57	30	0.926	/	/
	b004060	2A	780.97–784.79	50	0.934	*QYr.cim-2AL*	Zhang et al. (2022)
	b004312	2B	51.89–54.18	36	0.931	*QYr.sgi-2B.1*;* QYr.spa-2B*	Prins et al. (2011); Bokore et al. (2017)
	b005455	2B	766.61–770.98	66	1	*wmc332*	Christiansen et al. (2006)
	b005713	2B	804.91–809.15	76	0.926	*QYrpd.swust-2BL.4*	Zhou et al. (2022)
	b011539	5A	48.05–66.82	51	0.922	*QYr.gaas-5AL*	Bai et al. (2022)
	b011540	5A	66.85–105.92	85	0.959	*QYr.ifa-5A*	Buerstmayr et al. (2014)
	b012818	5B	499.26–510.69	102	0.946	*QYr.caas-5BL.1*	Lu et al. (2009)
	b014140	6A	19.95–22.85	27	0.938	*Yrq3*	Cao et al. (2012)
	b014220	6A	66.60–76.64	38	0.927	/	/
RMSD	b000322	1A	165.7–247.33	62	0.996	*QYr.hebau-1AL*	Gebrewahid et al. (2020)
	b003108	1D	494.81–497.25	42	0.972	/	/
	b003247	2A	14.82–17.96	40	0.980	*QYrtb.orz-2AS*	Vazquez et al. (2015)
	b003256	2A	18.30–19.91	54	0.996	*QYr.lrdc-2A.1*	Farzand et al. (2022)
	b010090	4A	650.49–656.83	42	0.990	/	/
	b010920	4B	538.96–553.02	40	0.963	*QYr.crc-4BL*	Rosa et al. (2019)
	b011005	4B	617.02–618.90	23	0.958	/	/
	b011680	5A	462.00–468.93	70	1	*QYr.hebau-5AL*	Gebrewahid et al. (2020)
	b011978	5A	582.69–583.70	18	0.958	*QYrsk.wgp-5AL*	Liu et al. (2019)
	b015121	6B	52.74–55.53	14	0.962	Close proximity *Yr36*	Fu et al. (2009)

^a^OP and RMSD indicate overall performance and root-mean-square deviation of stripe rust resistance, respectively; Hblock, haplotype block; Chrom, chromosome

^b^Physical position of each haploblock was based on IWGSC RefSeq v.2.1 (Tong et al. 2024b)

^c^The haploblock variance was calculated as the variance of haplotype effects at block, followed by being scaled to a 0–1 range (Voss-Fels et al. 2019b; Brunner et al. 2024)

^d^The closest linked markers of reported QTL were obtained from Tong et al. (2024a) and those loci with physical positions fallen into the identified haploblocks were seen as co-locations

### Parental selection combining OHS and SI approaches can advance genetic improvement for YR resistance and stability

**Fig. 6:**
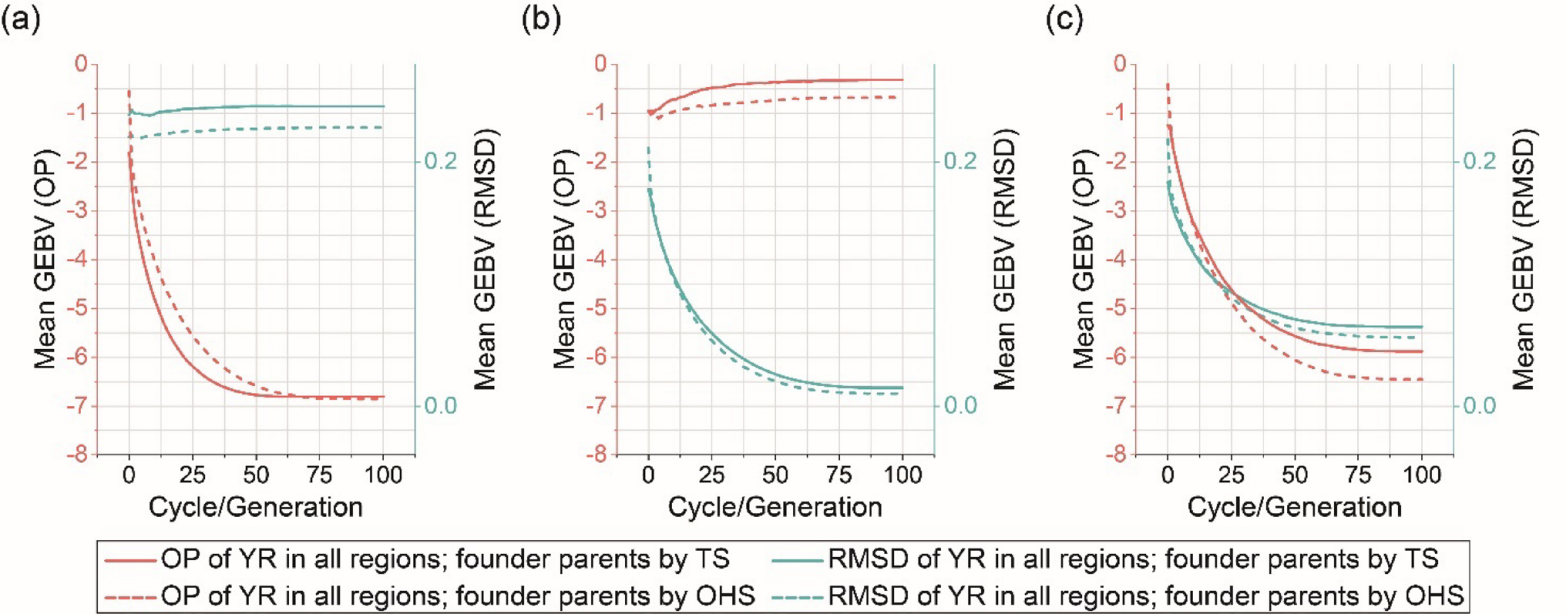
Simulation results of recurrent truncation selections starting with truncation selection (TS)-selected and optimal haplotype stacking (OHS)-selected founder parents for different selection objectives, including (a) overall performance (OP) of stripe rust resistance (YR); (b) root-mean-square deviation (RMSD) of YR resistance; and (c) selection index (SI) combining OP and RMSD. Plot shows the mean of GEBV in each cycle/generation in five replications, and 100 generations were set. The simulation runs in this figure use the parameters 10 progeny per cross and recurrent selection size of 25

The simulation results for different breeding and selection strategies across both environments (Australia and Ethiopia) are displayed in Fig. 6. It should be noted that lower values of OP and RMSD indicate improved YR resistance and better stability across environments, respectively. Selecting solely for the OP trait rapidly improved OP but had minimal impact on the RMSD trait (Fig. 6a), while selecting for RMSD slightly adversely affected OP (Fig. 6b). Using an index of OP and RMSD enhanced both traits (Fig. 6c), although the rate of improvement for either trait was not as high as when selecting for only one trait (OP or RMSD) individually. After 25 breeding generations, selection based on OP and OHS resulted in a mean GEBV for OP of approximately -5.5 (Fig. 6a), while selection based on SI and OHS yielded a mean GEBV around -5.0 (Fig. 6c). Additionally, selecting founder parents using OHS resulted in more long-term and rapid genetic gains (after many generations) compared to using TS, with the difference being slightly larger between TS and OHS when selecting on an index of OP and RMSD.

**Fig. 7:**
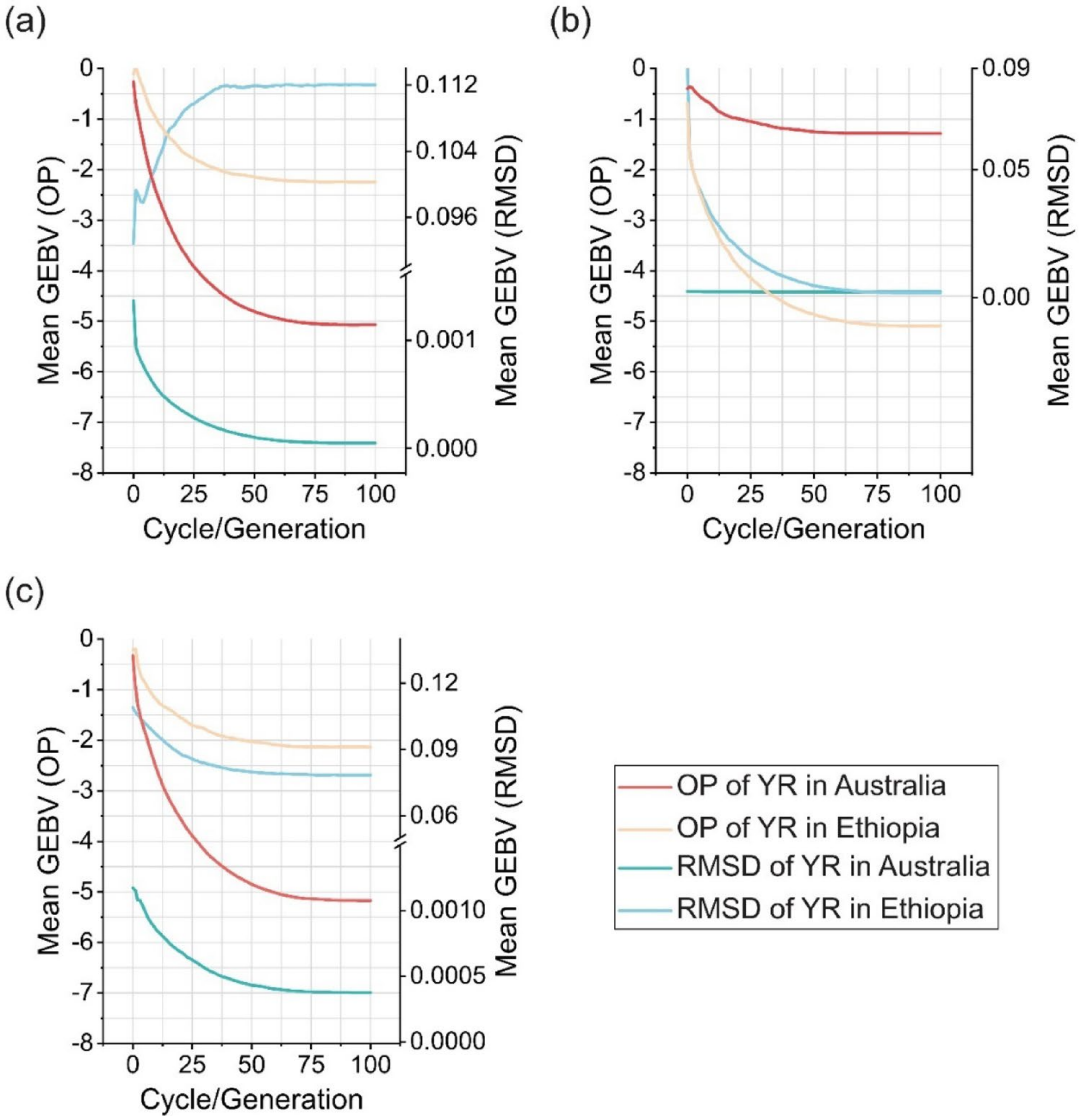
Simulation results of recurrent truncation selections starting with optimal haplotype stacking (OHS)-selected founder parents in different breeding regions. A selection index (SI) combining overall performance (OP) and root-mean-square deviation (RMSD) of stripe rust resistance (YR) is used for selection. The simulations run in different breeding regions, including (a) Australia; (b) Ethiopia; and (c) combined Australian and Ethiopian environments. Plot shows the mean of GEBV in each cycle/generation in five replications, and 100 generations were set. The simulation runs in this figure use the parameters 10 progeny per cross and recurrent selection size of 25

The simulations in Fig. 7 considered selection for SI in different breeding zones, including (a) Australia; (b) Ethiopia; and (c) combined Australian and Ethiopian environments. The OHS approach was employed to select founder parents. Selecting for SI in Australia improved both OP and RMSD of YR resistance in Australia, but RMSD in Ethiopia was predicted to be negatively impacted, while OP showed some improvement (Fig. 7a). Selection in Ethiopia showed a similar pattern, with no improvement in RMSD of YR resistance in Australia (Fig. 7b). Using SI across all regions for selection not only improved both OP and RMSD of YR resistance in all environments (Fig. 6c) but was also predicted to enhance both traits in each individual breeding zone (Fig. 7c). These results were also observed in Fig. S6, where TS was used for founder parent selection. Comparison of Fig. 7 with Fig. S6 showed that OHS achieved longer-term genetic improvement than TS for OP and RMSD of YR resistance in most breeding scenarios.

**Fig. 8:**
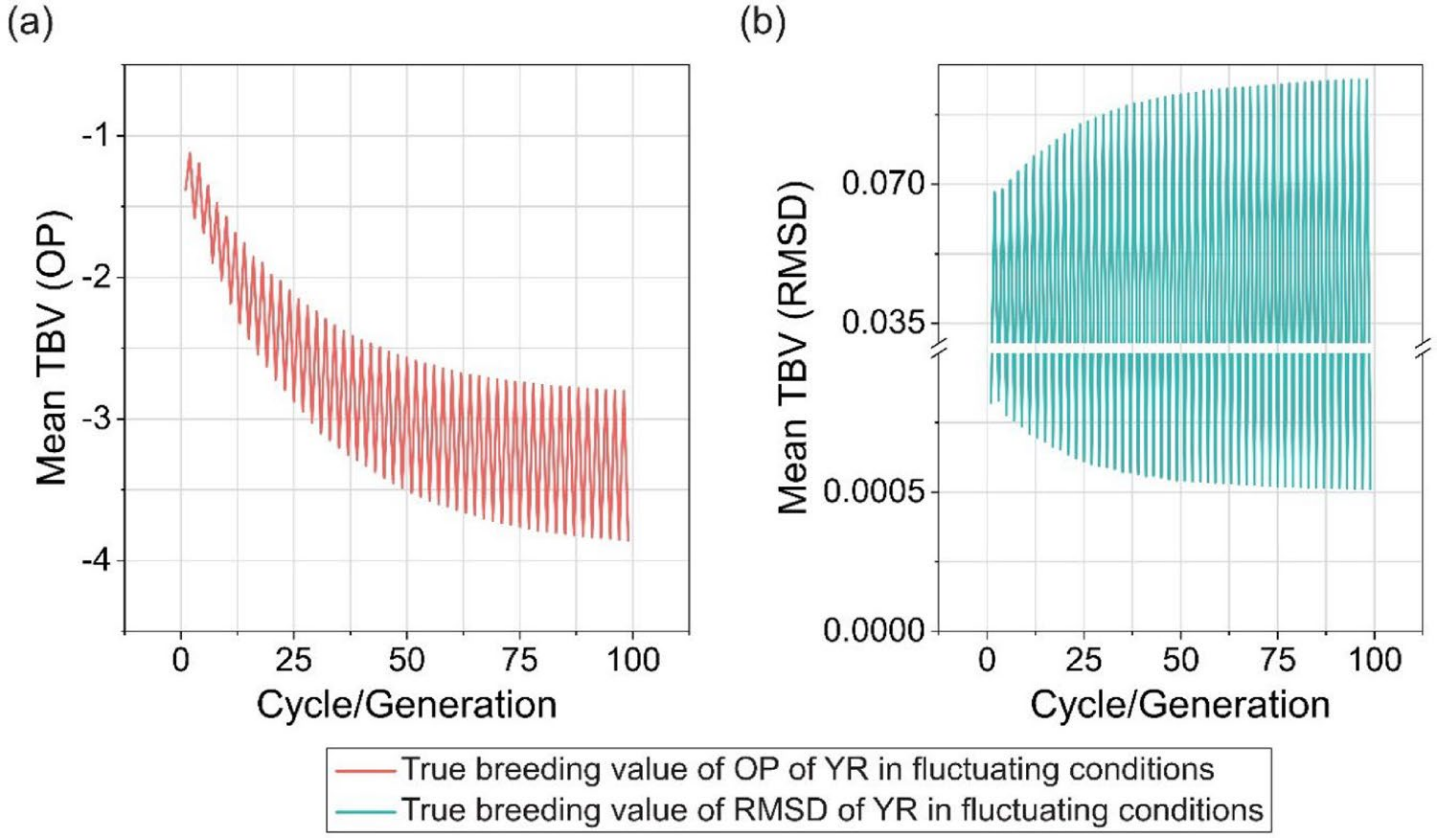
Simulation results of recurrent truncation selections starting with optimal haplotype stacking (OHS)-selected founder parents for overall performance (OP) of stripe rust resistance (YR) under fluctuating conditions, where Australian and Ethiopian environments alternate in 100 breeding generations. OP is the selection objective in the simulation. Plots show predicted changes of the mean of true breeding value (TBV) for (a) OP and (b) root-mean-square deviation (RMSD) of YR in each cycle/generation in five replications. The simulation runs in this figure use the parameters 10 progeny per cross and recurrent selection size of 25

**Fig. 9:**
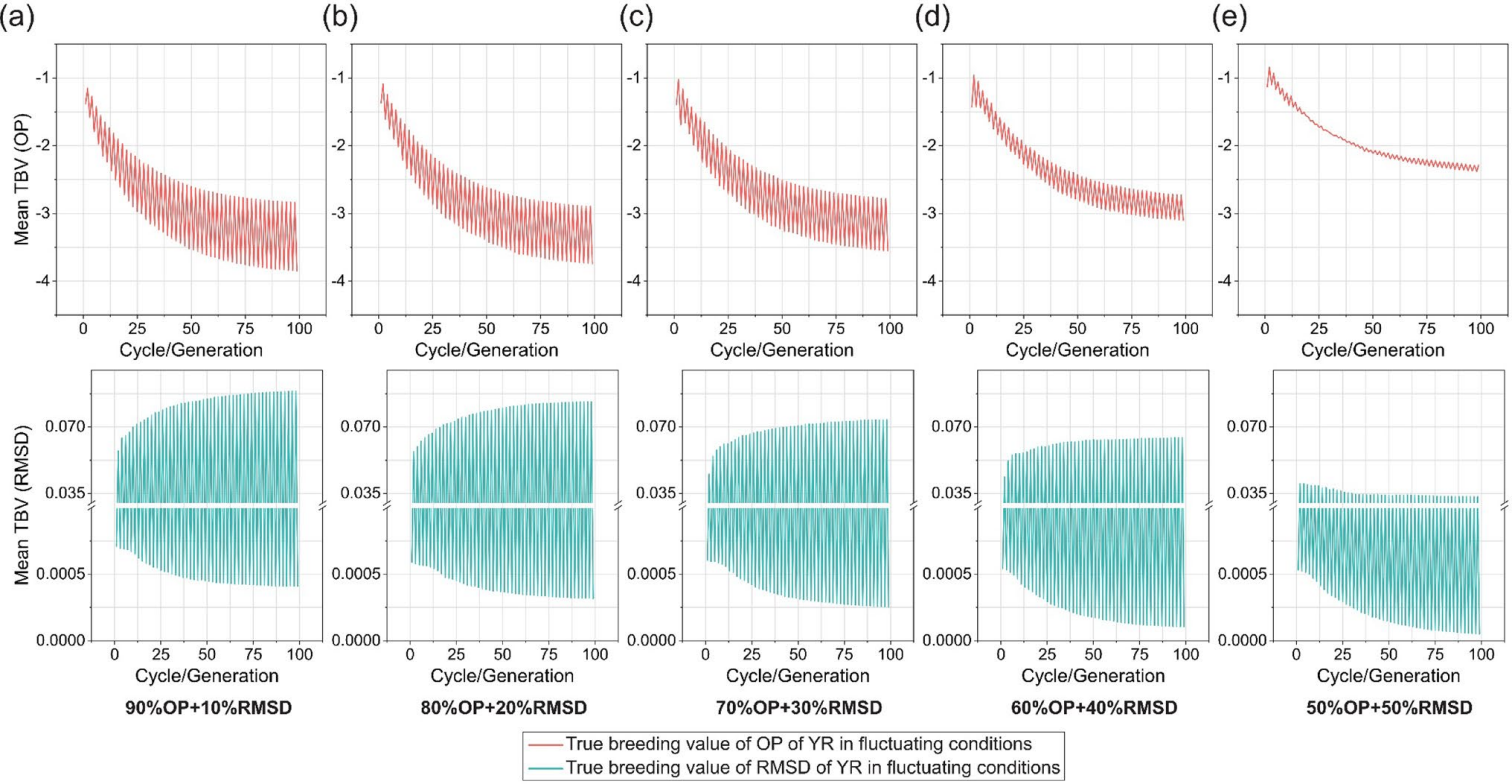
Simulation results of recurrent truncation selections under fluctuating conditions, where Australian and Ethiopian environments alternate in 100 breeding generations. Selection index (SI) combining overall performance (OP) and root-mean-square deviation (RMSD) of stripe rust resistance (YR) is used for selection, and a range of weight combinations of OP and RMSD are tested, including (a) 90%OP + 10%RMSD; (b) 80%OP + 20%RMSD; (c) 70%OP + 30%RMSD; (d) 60%OP + 40%RMSD; and (e) 50%OP + 50%RMSD. Plots show predicted changes of the mean of true breeding value (TBV) for OP (the top plots) and RMSD (the bottom plots) of YR in each cycle/generation in five replications. The simulation runs presented in this figure used the parameters of 10 progeny per cross and recurrent selection size of 25

To simulate the effects of fluctuating environmental conditions in one (hypothetical) location on OP and RMSD of YR resistance (for example year to year fluctuation caused by variable weather conditions or pathotype variation across seasons; Fig. S2), we assumed that the breeding selections occurred in a location with environmental conditions alternating between those of Australia and Ethiopia from year to year. In this scenario, selection for OP only resulted in a fluctuating but decreasing trend with increasing breeding generations, indicating an increase in genetic gain (Fig. 8a). RMSD decreased in the Australian environment, while it increased with generations in the Ethiopian environment (Fig. 8b). When a selection index with 10% or 20% on RMSD and the remainder on OP was used in this scenario, the RMSD score in the same breeding generation was lower compared to selection based solely on OP, while the OP score was similar (Figs. 8a, 9a, and 9b). However, RMSD did not improve for the Ethiopian environment with increasing breeding generations, where the RMSD score continued to exhibit an upward trend. Significant improvement in both RMSD and OP during the breeding process occurred when the weight of RMSD in the SI reached at least 50% (Fig. 9e). In this context, the rate of genetic gain for OP significantly decreased compared to selection focused solely on OP (Fig. 8a). As the weight on RMSD increased, the variability of performance decreased considerably.

## Discussion

This study utilised the Vavilov wheat diversity panel and MET data to analyse GEI patterns of YR resistance across Australia and Ethiopia, to reveal insights into the stability of YR resistance across environments. Haplotype-level identification was performed to understand the genetic basis of YR resistance and stability. Breeding selection strategies aiming to enhance both YR resistance and stability across different environments were evaluated through simulations, which provide new insights for breeders to increase genetic gain in resistance breeding programmes that target diverse and/or fluctuating environments.

### GEI analysis provides new insights into the stability of YR resistance across multiple environments

The disease response of plants and effectiveness of underlying resistance genes can be strongly affected by the external environment (Hickey et al. 2012; Pequeno et al. 2024). Understanding GEI patterns across multiple environments is crucial for the effectiveness of a breeding programme for disease resistance (Das et al. 2019; Sankar et al. 2021). In this study, we defined ‘environment’ as a composite factor for FA models, treating each unique location–year combination as a single environment. This approach not only captures the combined variation from location and year in the Ethiopian trials but also ensures consistency with the Australian trials, which were conducted at a single site. Although location and year were not modelled separately, integrating them into a composite environmental factor effectively reflects the GEI patterns under varying experimental conditions. We noted that the first-stage BLUEs were obtained from models with random block effects (in Ethiopian environments), which induce a nonzero covariance among the BLUEs. In the second-stage analysis, the use of a diagonal weighting matrix represents a simplification and may not fully capture the underlying covariance structure (Möhring & Piepho 2009). We also compared the FA model with the traditional three-factor LMM separating location and year effects. In terms of model fit criteria (e.g. AIC) (Table S4), the traditional model outperformed the more complex FA model, indicating that GEI effects could be studied using simpler models and analysis of variance. An additional hierarchical model that explicitly accounts for ‘country’ as a factor, with environments nested within country might capture the genetic covariance structure between environments more comprehensively, since high correlations were observed within Australian or Ethiopian environments in this study. Besides the FA model, an alternative approach for evaluating genotype performance and selecting stable cultivars in MET could be directly computing the marginal means for genotypes based on the traditional model. A wide range of variability of YR resistance was present in the Vavilov wheat collection across different environments, with significant cross-over GEI patterns indicated by differing genotype rankings across these environments. These results could be attributed to complex environmental factors associated with YR, such as field temperature, humidity, wind, and different pathotypes, which influence pathogen infection and plant response (Singh et al. 2011). The importance of these environmental conditions could be investigated using the FA model by correlating the covariates with the underlying latent factors from the model. Factors with strong correlations are inferred to drive the related model factors and GEI effects (Coast et al. 2022; Tolhurst et al. 2022). Due to the lack of detailed environmental data in this study, only the unknown/underlying factors and their rotated loadings from the FA model were used to group similar environments together, resulting in the definition of two breeding zones, Australia and Ethiopia. This seems reasonable, as Australian experiments were conducted in YR nurseries where plants were infected through artificial inoculation and under favourable weather conditions (e.g. high humidity). In Ethiopia, natural infections were caused by local and virulent pathotypes distinct from those in Australia. Therefore, it is speculated that differences in environmental conditions and/or pathotypes were major drivers in the significant GEI between Australia and Ethiopia, while a high degree of genetic correlation within Australian or Ethiopian environments indicated relatively low GEI. Characterising the key and specific environmental factors driving GEI is worth investigating in future studies.

Three strategies have been proposed to effectively handle GEI in breeding programmes (Eisemann et al. 1990). To reduce or leverage GEI, one can select genotypes that are well adapted to specific environmental groups (i.e. with high specificity) or stable genotypes that perform well across most environments (i.e. with broad adaptability) (Eisemann et al. 1990; Verma et al. 2024). When the breeding objective is to identify broadly adapted genotypes within a target population of environments, modelling environments as random might be preferred. In our MET model, we modelled GEI effects using a FA structure. We employed a quantitative method developed by Smith and Cullis (2018), which calculates two derivatives of the FA structure, OP and RMSD, to assess the average performance and stability of genotypes across environments. This method is expected to be routinely adopted in plant breeding, and a key advantage is that all measures are on the same scale as the trait, and OP and RMSD are independent, facilitating easy integration into a selection index (Smith and Cullis 2018). In this study, we attempted a simple inverse linear transformation to reconstruct the original phenotypic trait from OP. The estimated values of the original disease phenotype (YR) could be obtained through $\widehat{YR} = F{\Lambda }^{T}$, given that OP is derived from the first latent factor ($f_{1}$) and mean environmental loading ($\overline{\lambda }_{1} )$. However, FA model is a dimensionality reduction method, hence it cannot fully restore all original phenotypic information, i.e. $\widehat{YR}$ is only approximately equal to YR. Since the OP and transformation of OP retain only the most significant factor F_1_, the reconstructed phenotype may lack certain information. We evaluated the Pearson correlation coefficient between the reconstructed and real phenotypic values in the Australian environments (*r* = ~ 0.8, data not shown), which indicated most information was retained during the transformation. The Vavilov accessions selected for the best OP and/or RMSD have the potential to improve YR resistance and stability across diverse breeding regions (Table S6). Additionally, some genotypes that maximise the diversity of desirable haplotypes were selected using OHS (Table S7; discussed below). The majority of the Vavilov accessions are landraces that are unadapted to modern cultivated environments but serve as valuable donor parents offering resistance haplotypes, which can be transferred into elite wheat backgrounds through intercrossing and/or backcrossing in breeding programmes (Riaz et al. 2017; Jambuthenne et al. 2022). The accessions can also be used to develop new research populations for gene mapping and association studies to further elucidate YR resistance stability at the molecular level.

A simple MT-GBLUP model, where each environment was treated as a separate trait, was used to investigate whether the multi-trait model was comparable to the relatively complex FA model in terms of selecting resistant and stable genotypes. The GEBV from the multi-trait and the FA approaches were in good agreement across different breeding regions, except for YR resistance stability in Australia (Fig. 4). This was likely because Australian environments had much less GEI effect compared to Ethiopian and other environments, resulting in very small values of RMSD and corresponding GEBV for genotypes, as shown in Fig. S5. This led to another interesting finding: for the single-trait GBLUP (ST-GBLUP) model, the correlation between average GEBV and OP was very high in Australia but decreased significantly across other regions. This discrepancy arose because the FA model incorporated genetic effects including GEI, whereas the ST-GBLUP model evaluated genetic effects for each individual environment, excluding GEI. The FA and MT-GBLUP models are better at identifying wheat accessions with high resilience to YR resistance in varying environments with significant GEI.

A possible problem here is that OP is obtained as a BLUP from the FA model, and another round of GBLUP is further shrinking the shrinkage estimate (Holland & Piepho 2024). To address this concern, we have applied a deregression approach to compute the deregressed values of OP and assess the extent of shrinkage. We define shrinkage as the reduction in the BLUP estimate due to limited information, which can be approximated using the reliability coefficient (*r*^2^), i.e. $Deregressed OP_{i} = \frac{{OP_{i} }}{{r_{i}^{2} }}$. The reliability can be calculated as $r_{i}^{2} = 1 - \frac{{SE\left( {OP_{i} } \right)^{2} }}{{\sigma_{OP}^{2} }}$. Since ${\text{OP}}_{i} = { }\overline{\lambda }_{1} f_{1i}$, where $f_{1i}$ is the genotype score for the first latent factor, but $SE\left( {f_{1i} } \right)$ is not given in the FA model, we can use $SE\left( {BLUP_{GE} } \right)$ to approximate, i.e. $SE\left( {OP_{i} } \right) \approx SE\left( {BLUP_{GE} } \right)$. But in practice $SE\left( {OP_{i} } \right)$ is expected to be smaller, and the actual reliability (*r*^2^) of OP is likely underestimated by this approximation. $SE\left( {BLUP_{GE} } \right)$ can be calculated from the model’s predicted residual variance structure, i.e. $SE\left( {BLUP_{GE} } \right) = \sqrt {\frac{1}{{n_{e} }} \mathop \sum \limits_{i = 1}^{{n_{e} }} Var_{i} }$. As a result, we generated the distribution of approximated deregressed OP values and reliability (*r*^2^) of OP within the Vavilov panel (Fig. S4), and a mean reliability (i.e. shrinkage-adjusted *r*^2^) was greater than 0.802. For the vast majority of lines, the reliability was within a narrow range centred around this value of 0.802, indicating almost all lines received the same amount of (limited) shrinkage. Additionally, the Pearson correlation between OP and its deregressed values was 0.87 (data not shown). These results suggested that although OP is technically a BLUP derived from the FA model, it closely reflects the true genetic variability of each Vavilov genotype and thus exhibits high reliability. Our objective was to incorporate genome-wide marker information via a GBLUP model to calculate GEBV to accelerate prediction accuracy and genomic selection. The comparison between the FA-based approach and MT-GBLUP model also supports using OP as an effective input in assisting breeding decisions. Another potential problem is that the distribution of RMSD is right skewed as the input of the GBLUP model. Accordingly, it may be more appropriate to analyse log transformed RMSD, thereby better aligning with the assumptions of the GBLUP model. However, although the GBLUP model assumes that residuals are approximately normally distributed, some studies have shown that it might still provide robust predictions even when the phenotypic data do not strictly follow a normal distribution (Heslot et al. 2012; Crossa et al. 2017).

### The genetic basis of YR resistance and stability is revealed through haplotype characterisation

To elucidate the genetic basis of YR resistance stability, a haplotype-based mapping approach (Voss-Fels et al. 2019b; Villiers et al. 2024) was employed to assess the genetic effects of each haplotype segment on the wheat genome concerning OP and RMSD across environments. This approach contrasts with single-marker methods used in traditional GWAS, which can overestimate marker effects due to repeated counting of markers (Voss-Fels et al. 2019b). The haplotype-based approach focuses on the variance of effects for the block haplotypes and does not have a defined significance threshold. In this study, we identified only the top ten haploblocks with the highest variances and scaled variances exceeding 0.9 as the most likely to be associated with the trait. We also tried to define a significance level using a chi-square test. Under a quasi-infinitesimal model, the expected variance from a block would be the total genetic variance for the trait divided by the number of blocks. A chi-square test was performed as (observed variance for the block being tested – expected variance) ^2/expected variance, followed by comparing this to a chi-square test with one degree of freedom. This was extended to the top ten blocks by summing up the results for each individual block, then we compared it to a chi-square test with nine degrees of freedom at *P* < 0.05. However, the results showed that only b005455 departed significantly from the quasi-infinitesimal model (Table S5), indicating that the chi-square test approach we tried here is stringent, and significant haplotype blocks are only likely to capture a very small proportion of the variance. Hence this criterion is not suitable for selecting haplotypes to stack to create high performing genotypes. Note that a haploblock may encompass multiple causal genes or alleles responsible for the trait (Song et al. 2023).

Although OP and RMSD of resistance are independent traits, previous QTL mapping studies related to YR have reported nearly all the OP- and RMSD-associated loci identified in this study (Table 3). This suggests that the genetic basis of YR resistance stability may have been largely elucidated by studies focusing on mapping adult plant resistance (APR) genes in multi-environment field experiments (Vazquez et al. 2015; Bokore et al. 2017). A notable example is b015121, located in the vicinity of the race-nonspecific APR gene *Yr36* on chromosome arm 6BS, which encodes for Wheat Kinase START1 (Fu et al. 2009). This implies that broad-spectrum, durable resistance genes effective against multiple pathotypes may contribute to the genetic basis of the RMSD trait. Therefore, the haploblocks associated with RMSD are likely key breeding targets for developing foundational broad-spectrum resistance in wheat cultivars within breeding programmes.

In contrast, the lack of overlap between the haploblocks associated with OP and RMSD of YR resistance suggests that these traits may be governed by different genetic factors. Genotypes with desirable OP and RMSD of YR resistance exhibit broad adaptability across environments, while those with desirable OP but poor RMSD show high specificity. This indicates that OP is likely associated with both APR and all-stage resistance (ASR) genes, which are generally broad-spectrum and pathotype-specific resistance genes, respectively (Norman et al. 2023). The decision to target broad or environment-specific resistance largely depends on the breeding strategies employed (Verma et al. 2024). Overall, integrating GEI analysis to understand and dissect the genetic control of OP and RMSD of resistance in different environments will help develop resilient wheat cultivars capable of addressing future climate changes and evolving pathotypes. The combination of favourable haplotypes for OP and RMSD across environments might be an effective strategy to enhance resistance of wheat to multiple pathotypes.

### Breeding selection strategies to enhance YR resistance and stability across different environments

Based on the genetic effects of haplotype segments on the entire genome, several parental selection algorithms have been proposed, including OHS (Kemper et al. 2012), optimal population value (OPV) (Goiffon et al. 2017), and maximum haploid breeding value calculation (Müller et al. 2018). Villiers et al. (2024) found that OHS maintained greater genetic diversity and longer-term genetic gain than TS in a simulation study examining wheat yield using a commercial breeding dataset, provided the time horizon was sufficiently long. When initial parents in a recurrent selection breeding program were selected using OHS, there was a significant increase in both short-term and long-term genetic gain compared to parents selected via TS, enhancing the rate and maximum potential of yield improvement (Villiers et al. 2024). This finding is consistent with the results of the current study, which applied OHS for wheat disease resistance traits. Compared to TS, OHS achieved slightly greater long-term genetic gain for both OP and RMSD of YR resistance across breeding regions. Interestingly, this improvement was more pronounced when using the new index combining OP and RMSD for selection. SI is crucial for improving OP and RMSD simultaneously in the breeding process, as these traits are independent and distinct haploblocks are associated with each trait (discussed above). The breeding simulation also demonstrated that selecting only one trait did not lead to improvement in the other trait (Fig. 6). Through simulation, we also showed that selection for SI in Australia could not improve RMSD of YR resistance in Ethiopia, and vice versa, selection in Ethiopia was predicted not to affect RMSD in Australia. This result may be attributed to different genetic factors responsible for resistance stability in Australia and Ethiopia. However, selection based on SI in combined regions achieved genetic gains for both OP and RMSD of YR resistance in each individual region as well. This implies that the favourable resistance haplotypes (i.e. haplotypes reducing disease scores within high-variance haploblocks) identified in combined environments could be effectively against both Australian and Ethiopian pathotypes. These favourable haplotypes are valuable for breeding applications and can be rapidly and largely stacked using haplotype-based GS algorithms, such as OHS, to enhance YR resistance. Alternatively, breeder-friendly markers (e.g. KASP markers) can be developed in marker-assisted selection to purposefully introduce large-effect haplotypes into breeding materials (Xu et al. 2017). For multi-trait parental selection, Bayesian statistical strategies also provide a powerful alternative to traditional truncation selection methods (Villar-Hernández et al. 2021, 2024). For instance, the Multi-trait Parental Selection (MPS) R package integrates Bayesian optimization with genomic selection to efficiently balance genetic gain across multiple traits, even in the presence of negative correlations or missing data (Villar-Hernández et al. 2024).

The Vavilov accessions selected by OHS for SI in combined environments, listed in Table S7, are presumed to have relatively distant genetic relationships (Villiers et al. 2024), and therefore can maximise diversity for resistance haplotype segments. They differed significantly from genotypes selected based on truncation selection (Table S6), which possibly had more shared resistance haplotypes. The selected Vavilov accessions have potential utility in future resistance breeding programmes, depending on specific breeding objectives for different environments.

A range of weight combinations of OP and RMSD for SI were tested in breeding simulations under fluctuating conditions, where environmental characteristics alternated from year to year between those of Australia and Ethiopia. Simulated Ethiopian environments consistently had higher values of OP and RMSD than Australian environments, possibly due to more conducive conditions for YR occurrence in Ethiopia. A small weight for RMSD (10 or 20%) in SI effectively improved YR resistance stability under fluctuating conditions compared to selecting only for OP. The results highlight the importance of incorporating RMSD as a breeding target under variable conditions. An exciting outcome of including RMSD in the selection index was decreased variability of performance across years. In practice this would be attractive to wheat growers, as variability of performance can be difficult to manage.

While the simulations provide useful insights into selection methods, scenario (3) did not explicitly account for potential environmental effects or gene-by-environment interactions. Future studies should integrate additional environmental variables, such as detailed weather and pathotype features, into GEI analysis and breeding simulations to enable more accurate predictions (Resende et al. 2024). Additionally, it is highly recommended to include not only the Vavilov collection but also elite wheat cultivars in the GEI analysis and use them as recurrent parents in more complex, real-world breeding simulations. Overall, this study advances the understanding of the GEI of YR resistance, offering novel insights into resistance stability and providing selection strategies for achieving rapid and stable genetic gains for YR resistance across diverse, and fluctuating global environments.

## Supplementary Information

Below is the link to the electronic supplementary material.

Supplementary file1 (DOCX 740 kb)

Supplementary file2 (XLSX 40 kb)

## Data Availability

The data presented in the study is included in the article/supplementary materials; further inquiries can be directed to the corresponding authors.
