# Supplementary material for: Haplotype stacking to improve stability of stripe rust resistance in wheat
Source: Theor Appl Genet. 2025 Oct 6;138(11):267. doi: 10.1007/s00122-025-05045-0 (PMC12500776; doi:10.1007/s00122-025-05045-0)
Supplement: Supplementary file 1 — Supplementary file1 (DOCX 740 kb) [file 122_2025_5045_MOESM1_ESM.docx]

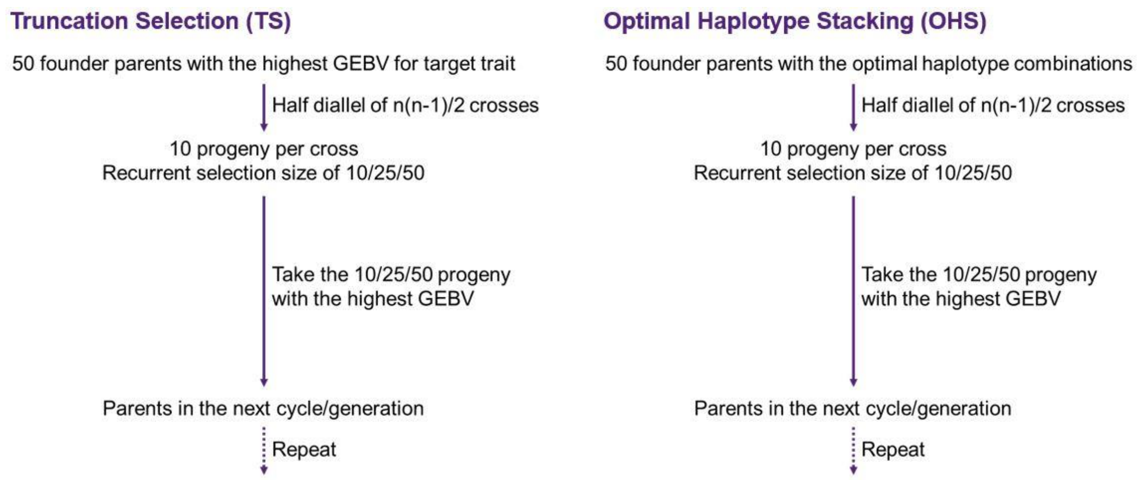

Figure S1 Structure of the breeding simulation experiment. Two approaches, truncation genomic selection (TS) and optimal haplotype stacking (OHS), were used to select the founder parents, respectively. 100 breeding cycles/generations were carried out as a long timeframe.

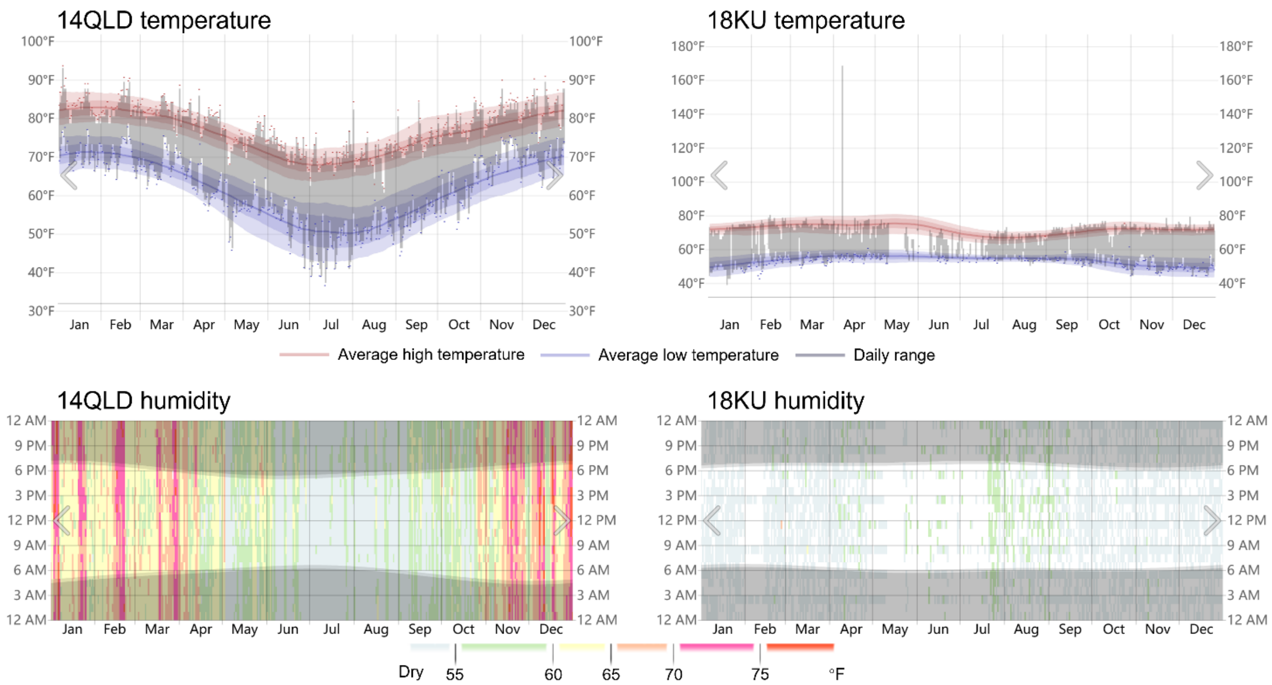

Figure S2 Weather data for experimental sites in Queensland and Kulumsa during one experimental year. The above shows the daily range of reported temperatures (gray bars) and 24-hour highs (red ticks) and lows (blue ticks), placed over the daily average high (faint red line) and low (faint blue line) temperature, with 25th to 75th and 10th to 90th percentile bands. The bottom shows the hourly reported humidity comfort level, categorized by dew point, and the shaded overlays indicate night. 14QLD, experimental site in Queensland in year 2014; 18KU, experimental site in Kulumsa in year 2018. Data was sourced from https://weatherspark.com/.

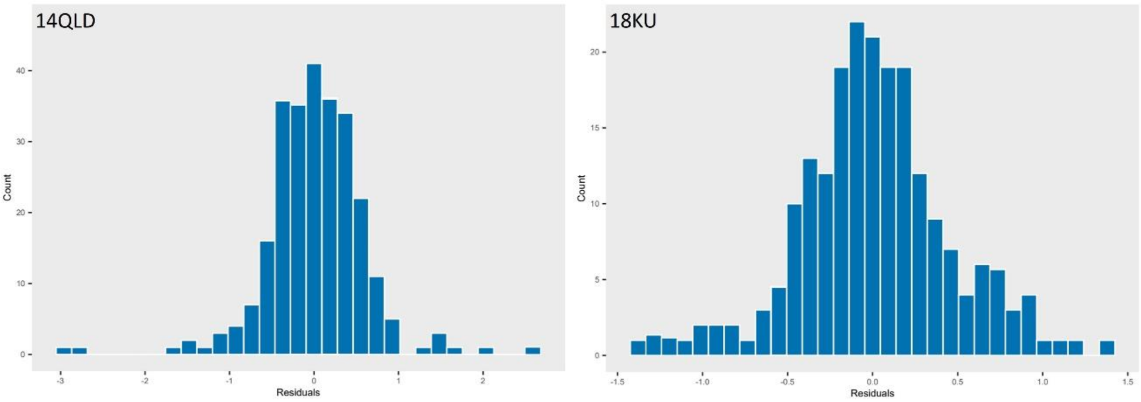

Figure S3 Residual plots of fitting linear mix models to disease data for single-environment analysis. 14QLD, experimental site in Queensland in year 2014; 18KU, experimental site in Kulumsa in year 2018.

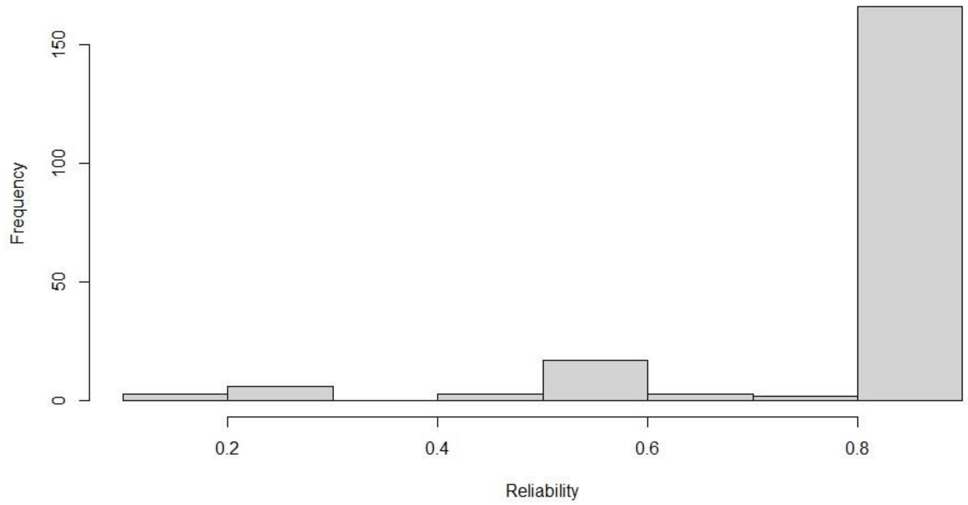

Figure S4 Reliability (r^2) of overall performance (OP) of stripe rust resistance across environments for the Vavilov wheat accessions. Reliability was estimated to reflect the shrinkage extent and accuracy of OP derived from the factor analytic model.

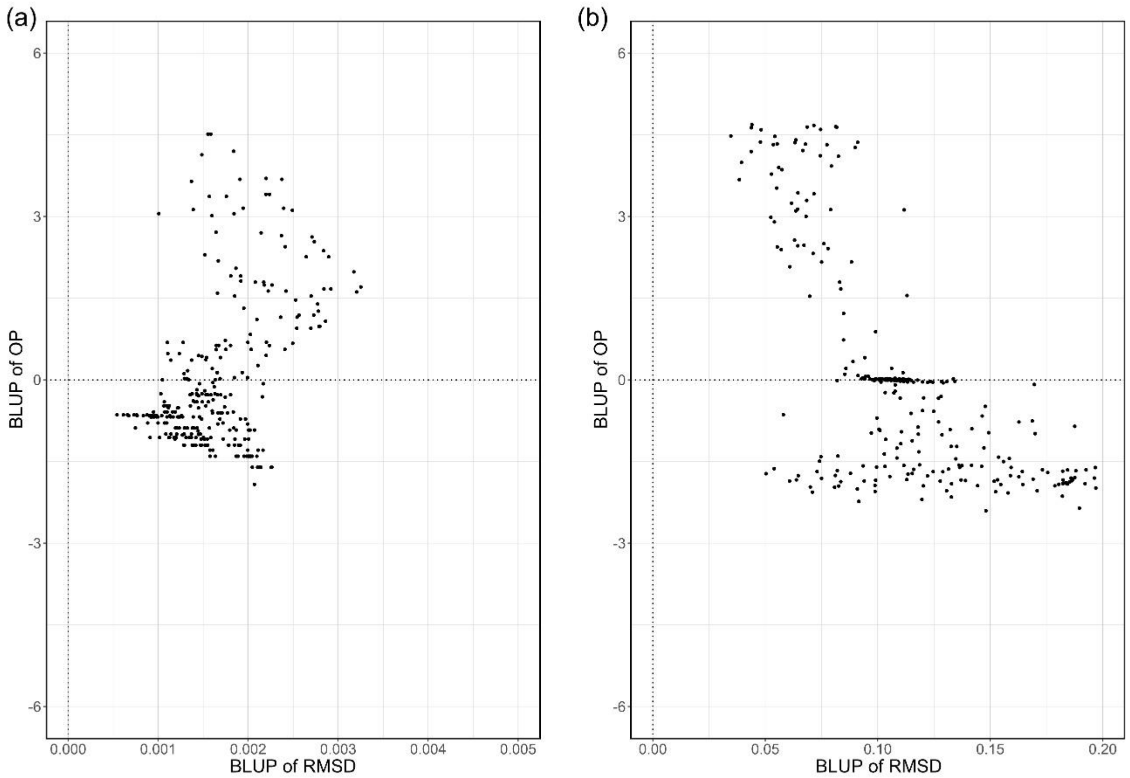

Figure S5 Overall performance (OP) versus root-mean-square deviation (RMSD) for stripe rust (YR) resistance of 295 Vavilov wheat accessions evaluated in (a) Australia and (b) Ethiopia.

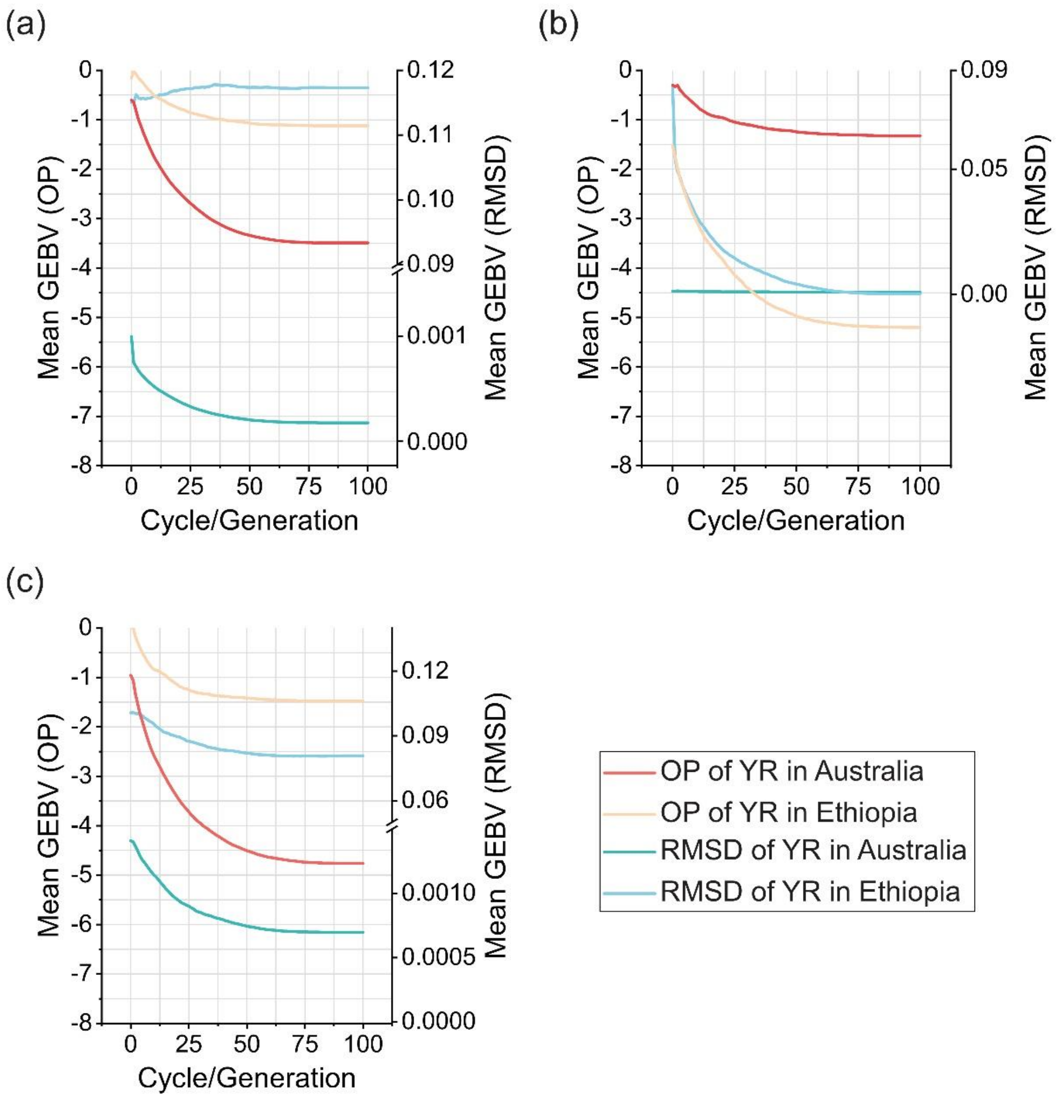

Figure S6 Simulation results of recurrent truncation selections starting with truncation selection (TS)-selected founder parents in different breeding regions. A selection index (SI) combining overall performance (OP) and root-mean-square deviation (RMSD) of stripe rust resistance is used for selection. The simulations run in different breeding regions, including (a) Australia; (b) Ethiopia; and (c) combined Australian and Ethiopian environments. Plot shows the mean of GEBV in each cycle/generation in five replications, and 100 generations were set. The simulation runs in this plot use the parameters 10 progeny per cross and recurrent selection size of 25.
